# Sodium-glucose co-transporter-2 inhibitors in the intensive care unit setting: do we really need sodium increase, especially in sepsis? Reply

**DOI:** 10.1186/s13054-023-04513-7

**Published:** 2023-06-08

**Authors:** Johan Mårtensson, Rinaldo Bellomo

**Affiliations:** 1 Department of Physiology and Pharmacology, Section of Anaesthesia and Intensive Care, Karolinska Institutet, Stockholm, Sweden; 2 Department of Perioperative Medicine and Intensive Care, Karolinska University Hospital, 171 76 Stockholm, Sweden; 3 Department of Intensive Care, Austin Health, Melbourne, VIC Australia; 4 Australian and New Zealand Intensive Care Research Centre, Monash University, Melbourne, VIC Australia; 5 Department of Critical Care, Melbourne University, Melbourne, VIC Australia; 6 Department of Intensive Care, Royal Melbourne Hospital, Melbourne, Australia

Dear Editor,

We thank Dr Patoulias for highlighting the importance of sodium levels in patients treated in the intensive care unit (ICU) and the potential implications of our study on the use of empagliflozin in this setting [1, 2].

We would like to emphasize that our study aimed to investigate the safety and efficacy of empagliflozin, a sodium-glucose co-transporter-2 (SGLT-2) inhibitor, in subjects with underlying type 2 diabetes mellitus admitted to the ICU, with a particular focus on changes in sodium levels as an exploratory outcome [2]. Our intention was not to intentionally increase sodium levels, but rather to observe and document any changes associated with the use of empagliflozin.

As Dr Patoulias correctly points out [1], empagliflozin therapy was associated with increasing sodium levels reaching a median (IQR) maximum level of 149 (142–155) mmol/l within a week. Indeed, such levels have been associated with harm in large observational studies [3]. We appreciate that hypernatremia is a potential complication to SGLT2 therapy, which must be considered in the design of future ICU trials involving this drug class. Whether the concomitant use of electrolyte-free drug diluents and maintenance fluids attenuate the risk of hypernatremia in this setting remains to be seen.

In response to Dr Patoulias’ observation regarding the baseline presence of sepsis, we agree that performing a subgroup analysis based on sepsis status could provide additional insights into the effects of empagliflozin in this specific patient population. Four control group patients and ten treatment group patients were admitted with sepsis. Sodium levels in the control group increased from 134 (IQR 128–137) mmol/l to 145 (IQR 140–153) mmol/l. In the treatment group, sodium levels increased from 137 (IQR 133–150) mmol/l to 148 (IQR 138–155) mmol/l. Although both groups experienced increases in sodium levels, the magnitude of the increase was notably smaller in the empagliflozin treatment group. Given the limited statistical power and lack of data on administered fluid volumes and their electrolyte compositions, this additional finding should be interpreted with caution.

## Data Availability

Not applicable.

## References

[1] Patoulias D (2023). Sodium-glucose co-transporter-2 inhibitors in the intensive care unit setting: do we really need sodium increase, especially in sepsis?. Crit Care.

[2] Mårtensson J, Cutuli SL, Osawa EA, Yanase F, Toh L, Cioccari L (2023). Sodium glucose co-transporter-2 inhibitors in intensive care unit patients with type 2 diabetes: a pilot case control study. Crit Care.

[3] Waite MD, Fuhrman SA, Badawi O, Zuckerman IH, Franey CS (2013). Intensive care unit-acquired hypernatremia is an independent predictor of increased mortality and length of stay. J Crit Care.

